# Catalytic Oxidation of Soot on a Novel Active Ca-Co Dually-Doped Lanthanum Tin Pyrochlore Oxide

**DOI:** 10.3390/ma11050653

**Published:** 2018-04-24

**Authors:** Lijie Ai, Zhongpeng Wang, Chenchen Cui, Wei Liu, Liguo Wang

**Affiliations:** School of Resources and Environment, Key Laboratory of Water Resources and Environmental Engineering in Universities of Shandong, University of Jinan, 336 Nanxinzhuang West Road, Jinan 250022, China; ailj20160202@163.com (L.A.); 15053102308@163.com (C.C.); stu_liuw@ujn.edu.cn (W.L.); chm_wanglg@ujn.edu.cn (L.W.)

**Keywords:** pyrochlore, oxygen vacancy, soot removal, catalytic oxidation

## Abstract

A novel active Ca-Co dually-doping pyrochlore oxide La_2−x_Ca_x_Sn_2−y_Co_y_O_7_ catalyst was synthesized by the sol-gel method for catalytic oxidation of soot particulates. The microstructure, atomic valence, reduction, and adsorption performance were investigated by X-ray powder diffraction (XRD), scanning electron microscope (SEM), Fourier-transform infrared spectroscopy (FT-IR), X-ray photoelectron spectroscopy (XPS), H_2_-TPR (temperature-programmed reduction), and in situ diffuse reflection infrared Fourier transformed (DRIFTS) techniques. Temperature programmed oxidation (TPO) tests were performed with the mixture of soot-catalyst under tight contact conditions to evaluate the catalytic activity for soot combustion. Synergetic effect between Ca and Co improved the structure and redox properties of the solids, increased the surface oxygen vacancies, and provided a suitable electropositivity for oxide, directly resulting in the decreased ignition temperature for catalyzed soot oxidation as low as 317 °C. The presence of NO in O_2_ further promoted soot oxidation over the catalysts with the ignition temperature decreased to about 300 °C. The DRIFTS results reveal that decomposition of less stable surface nitrites may account for NO_2_ formation in the ignition period of soot combustion, which thus participate in the auxiliary combustion process.

## 1. Introduction

The diesel engine has been widely used in the transport vehicle market for decades due to its inherent high thermal efficiency, low fuel consumption, and durability. However, particulate matter (PM) from diesel engine emissions is gradually evolving to a serious threat to human health and the global environment [1,2,3,4]. Increasingly stringent emission regulations require solutions based on appropriate post-processing technology and improvements to the engine. The combination of diesel particulate filter (DPF) and the catalytic combustion of soot appears to be a promising after-treatment technique for soot removal, where soot is first captured by DPF and then removed by catalytic combustion [5]. Up to now, a variety of catalysts have been widely investigated and applied to soot removal, including noble metal [4,6], alkaline metal oxides [6,7], transition metal oxides [8,9], as well as mixed metal oxide [10] materials. Although these catalysts have certain advantages in catalyzing soot combustion, they are still constrained by some drawbacks. For example, noble metals have excellent activity at low temperatures, but they are expensive and less stable at high temperatures. Alkali metal catalysts also have good activity but are easily lost at high temperatures. Consequently, there is still substantial interest in the development of low-cost, better-performing, and more durable soot oxidation catalysts.

Pyrochlore oxides are a family of new inorganic functional material with open structure, which show excellent chemical adjustable denaturation and thermal durability in the catalytic reaction. In recent years, stannate pyrochlore has been well applied to the fields of CH_4_ oxidation and NO oxidation due to its superior physical and chemical properties [11,12]. Pyrochlores always have the empirical formula A_2_B_2_O_7_, where A generally designates a rare-earth with a +3 valence state forming a tetrahedron around the oxygen ions and B a transition metal cation with a +4 valence state combined with oxygen to form [BO_6_] octahedrons. Similar to perovskite, this structure can tolerate a certain degree of cation and anion vacancies and also a mixed valence state of transition with metal ions while maintaining stable. The A-site of the pyrochlore structure is mainly responsible for the thermal stability, which once substituted with the alkali metal or alkaline earth metal the valence and distribution of the B-site ions may be changed. Meanwhile, some compensating oxide ion vacancies would be introduced due to the lower charge of doping ions such as Sr^2+^, Y^3+^ and Ca^2+^ to further enhance gaseous oxygen adsorption and oxygen-ion migration. It indicated that the “A” ions indirectly affect the catalytic activity [13]. In addition, the B-site ions are usually “active” ions, mainly responsible for the activity of the catalyst. Transition-metal cations such as Cu^2+^, Co^3+^, Fe^3+^ and Mn^3+^ have always been introduced as dopants into B-site of pyrochlore lattice, and these metals themselves may present additional redox activity due to their accessible reducing capability [14]. 

In the recent studies it was found that cobalt incorporation can greatly improve redox capacity of La_2_Sn_2_O_7_ to facilitate CH_4_ oxidation and NO oxidation [14,15]. Besides, metal-doped pyrochlore has also been reported in catalyzing soot combustion. Nevertheless, there are few studies that can provide sufficient data on the A, B-site double doped pyrochlore for soot removal. Alkali metal and alkaline-earth metal oxides have been claimed to bring stupendous benefit for soot oxidation in many reports [16,17]. Luca Lietti et al. also proposed the theory of electrodeposition of alkali and alkaline earth metals to the soot oxidation, which inspired us to study the substitution of Ca to the A site of pyrochlore in this study [7]. In order to investigate the effect of the single and double substitution in A/B sites of La_2_Sn_2_O_7_ for catalyzing soot combustion, a series of mixed oxides La_2−x_Ca_x_Sn_2−y_Co_y_O_7_ were synthesized, characterized, and tested in this work. The structure-activity relationship of the catalysts was constructed by various means such as scanning electron microscope (SEM), X-ray photoelectron spectroscopy (XPS), temperature-programmed reduction (TPR) and temperature programmed oxidation (TPO). In particular, the in situ DRIFT technique was employed with individual catalysts in the presence of NO + O_2_, to explore the intermediate species that produced by the NO_2_ auxiliary combustion process.

## 2. Experimental

### 2.1. Catalyst Preparation

La_2−x_Ca_x_Sn_2−y_Co_y_O_7_ (x = 0, 0.4; y = 0, 0.2) pyrochlore oxides were prepared by CTAB-assisted sol-gel method [18]. The reagents used were analytically pure and no further purification was required. The stoichiometric amounts of SnCl_4_·5H_2_O and Co (NO_3_)_3_·6H_2_O were dissolved in 30 mL of deionized water and then heated the mixed salt solution to 80 °C. An appropriate amount of 1 mol/L sodium hydroxide solution was added dropwise to the above solution to form a white precipitate accompanied by constant magnetic stirring to ensure the salt ions were completely precipitated. In addition, an appropriate amount of La (NO_3_)_3_·6H_2_O and CaCl_2_ were dissolved in the same manner and a white slurry was formed. Afterwards, the two slurries were slowly mixed together, accompanied by continuous stirring. The pH of the mixed solution was adjusted to 10 with 1 mol/L NaOH solution. A certain amount of cetyltrimethyl ammonium bromide(CTAB) with a ratio 1:1 to metal cations was rapidly dissolved in 50 mL of deionized water by sonication, followed by addition to the above-mentioned precipitation mixed solution, and the pH was controlled around 12.5. The mixture was then stirred continuously in a water bath for 5 h at 80 °C and retained at 80 °C for 24 h. Afterwards, the supernatant was decanted and the solid residue was filtered and washed several times with water and ethanol by crossing. The achieved wet cake was dried overnight at 110 °C and then calcined at 900 °C for 6 h in an air atmosphere to get the finalized catalysts. For convenience, the obtained catalysts were denoted as LS (x = y = 0), LCS (x = 0.4, y = 0), LSC (x = 0, y = 0.2), LCSC (x = 0.4, y = 0.2), respectively.

### 2.2. Catalyst Characterization

XRD patterns were measured on a BRUKER-AXS D8 Advance X-ray Diffractometer (Bruker, Billerica, MA, USA) using Cu Kα radiation (λ = 0.15418 nm). The X-ray tube was operated at 40 kV and 40 mA. The data of 2θ from 20° to 80° were recorded at 0.02° intervals with a scanning velocity of 6°/min.

The surface morphology was confirmed by a Quanta FEG250 SEM instrument (FEI, Houston, TX, USA) using an accelerating voltage of 10 kV. In order to improve the electrical conductivity, the sample was previously sprayed a very thin gold layer before scanning.

Specific surface area and pore distribution information of the prepared catalysts were derived from the corresponding nitrogen adsorption isotherm conducted on a Micromeritics ASAP 2020 surface area analyzer (Mcromeritics Instruments, Norcross, GA, USA). All the samples were degassed at 300 °C for 5 h under vacuum prior to measurement.

FT-IR spectroscopy were recorded on a Bruker Tensor 27 spectrometer (Bruker, Ettlingen, Germany) over 400–4000 cm^−1^ after 16 scans at a resolution of 4 cm^−1^. The catalysts were prepared in the form of pressed wafers (ca. 1% sample in KBr).

H_2_-Temperature Programmed Reduction (H_2_-TPR) measurements were performed in a fixed-bed quartz reactor connected with a thermal conductivity detector (TCD) (Lunan Ruihong Chemical Instrument Corporation, Tengzhou, China) in series to record responses. Prior to each experiment, the 50 mg sample (40–80 mesh) was pretreated in N_2_ (50 mL/min) for 30 min to remove physisorbed water. After cooling to RT, it was then switched to an H_2_ (35 mL/min) gaspath and the temperature was raised to 900 °C at a rate of 10 °C/min.

XPS analysis was conducted on a Thermo Scientific Escalab 250Xi X-ray photoelectron spectrometer (Thermo Fisher Scientific, Waltham, MA, USA) equipped with a non-monochromatic Al Kα X-ray source. Correction of the energy shift due to the static charging of the samples was finished using C 1s peak of contaminant carbon (B.E. = 284.6 eV) as standard.

### 2.3. Catalytic Activity Measurement

Catalytic activity for soot combustion was investigated by TPO reaction in a continuous fixed-bed tubular quartz (i.d. = 10 mm) system. Printex-U (Degussa, Germany) was used as the model soot. Its average particle size is 25 nm and the specific surface area is 93.5 m^2^/g. Elemental analysis showed that its carbonaceous nature was 90.43 wt % C, 1.09 wt % H, 0.17 wt % N, and 0.51 wt % S. Volatility was detected to be about 5 wt % and desorbed at about 200 °C (by thermogravimetric analysis, TGA/DSC1/1600 HT, METTLER TOLEDO, ‎Greifensee‎, Switzerland). The soot and catalyst powders were mixed in a 1/9 weight ratio and grinded for “tight contact” conditions. A 50 mg soot/catalyst mixture was used for each trial. Prior to the experiment, the sample was pretreated under He flow (50 mL/min) maintaining at 200 °C for 30 min. After cooling to RT, the catalytic oxidation was carried out in 100 mL/min flow of 5 vol % O_2_/He or 1000 ppm NO/5% O_2_/He at 4 °C/min up to 700 °C. The effluent composition was analyzed by a linked on-line chromatograph with FID detector (GC) (SP-6890, Shandong Lunan Ruihong Chemical Instrument Corporation, Tengzhou, China) and a chemiluminescence NOx analyser (Model 42i-HL, Thermo Fisher Scientific, Waltham, MA, USA). Characteristic temperature (T_5_, T_50_, T_90_) at the conversion of 5%, 50% and 90% and the CO_2_-to-CO_x_ concentration ratio (S_CO2_) at the outlet were used to evaluate the catalyst activity by the TPO profiles.

NO oxidation on the La_2−x_Ca_x_Sn_2−y_Co_y_O_7_ (x = 0, 0.4; y = 0, 0.2) oxide catalysts was carried out in a fixed-bed quartz flow reactor using a 50 mg sample (40–80 mesh). The catalyst was pretreated in a flow of helium at 500 °C for 30 min and then cooled to RT followed by introduction of a mixed flow of 1000 ppm NO and 5 vol % O_2_ at a rate of 100 mL/min to begin test. The NO_x_ concentration was monitored by a chemiluminescence NO_x_ analyser (Model 42i-HL, Thermo Electron Corporation, Waltham, MA, USA) tracking in the experiment.

### 2.4. In Situ DRIFTS Experiments

The in situ diffuse reflection infrared Fourier transformed (DRIFT) experiments were carried out on a Nicolet iS50 spectrometer (Thermo Fisher Scientific, Waltham, MA, USA) equipped with a temperature-controllable diffuse reflection chamber and a high sensitivity MCT detector. The sample powder was purged in situ under a He stream flow at 50 mL/min with a temperature 500 °C for 30 min, and then cooled down to RT, simultaneously, the background spectrum of sample was collected at every testing temperature. Then a gas mixture of 1000 ppm NO/5%O_2_/He was fed at a flow rate of 100 mL/min, accompanied by raising the temperature to 500 °C and remaining in 500 °C for 30 min. All the spectra were determined by accumulating 16 scans at a resolution of 4 cm^−1^ as a function of temperature at a heating rate of 10 °C/min.

## 3. Results and Discussion

### 3.1. XRD Analysis

**Figure 1:**
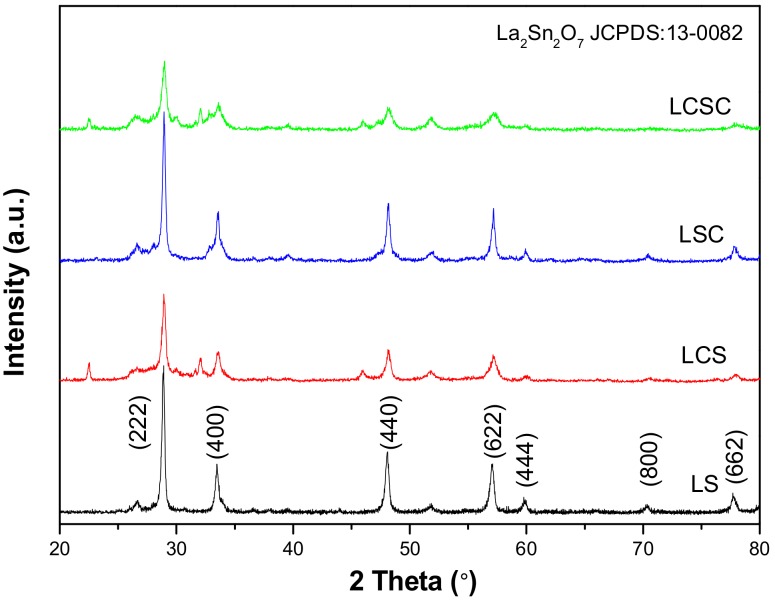
X-ray diffraction (XRD) patterns of La_2−x_Ca_x_Sn_2−y_Co_y_O_7_ pyrochlore oxides.

XRD characterization was conducted to identify the phase composition of the catalysts. Figure 1 gives the XRD patterns of the four pyrochlore oxides. All of the samples show obvious diffraction peaks at 2θ value of 28.9°(222), 33.5°(440), 48.1°(622), and 57.0°(400), which are typical diffraction patterns of the face-centered cubic structure La_2_Sn_2_O_7_(JCPDS 13-0082). Meanwhile, two small diffraction peaks at 26.6° and 51.7° are presented for all the samples, which can be assigned to the SnO_2_ formed due to an incomplete precipitation process. In addition, for the catalysts LCS and LCSC, a very weak diffraction signal at 2θ = 22.5°, 32.11° and 45.9° can be detected, suggesting that a small amount of CaSnO_3_ may be present. However, for the Co substituted samples, no other diffraction peaks pertaining to individual transition metal oxides can be detected. This well demonstrates that A-site substitution may lead to the formation of tiny separated phases, whereas B-site doping allows the doping ions to be highly dispersed into the crystal lattice, as reported previously [14].

The lattice parameter calculated from the Debye-Scherrer equation and the main peak position in the XRD patterns of the catalysts are summarized in Table 1. The obtained lattice parameters of three modified samples are smaller than that of pure La_2_Sn_2_O_7_ and the peak of crystal plane (222) slightly shifts to a higher 2θ angle. According to Bragg’s law, it means the lattice contraction and the decrease in d-spacing as a result of the substitution of larger La^3+^ (1.06 Å) and Sn^4+^ (0.69 Å) with smaller Ca^2+^ (0.99 Å) and Co^3+^ (0.63 Å) cations in the crystal structure, which indicates that the Ca^2+^/Co^3+^ ions are incorporated into the crystal lattice as expected [6]. In addition, a significant broadening of the peak width is observed in the Ca-containing samples, indicating the occurrence of a more defective lattice with smaller crystallite size. The mean crystallite sizes of all oxides are also shown in Table 1. It can be found that with the introduction of Ca, the grain size is significantly reduced, revealing that Ca inhibits the crystal growth of pyrochlore.

### 3.2. Catalyst Morphologies

**Figure 2:**
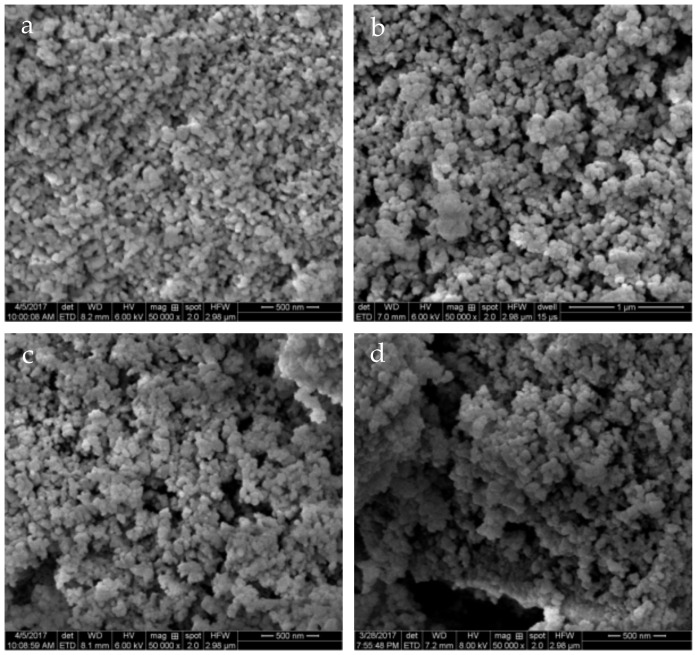
Scanning electron microscope (SEM) images of La_2−x_Ca_x_Sn_2−y_Co_y_O_7_ catalysts: (**a**) LS; (**b**) LCS; (**c**) LSC; (**d**) LCSC.

As a solid-solid-gas reaction, the morphology of the catalyst strongly influences the catalytic oxidation of soot. In general, catalysts with high specific surface area and large pores can better contact with soot, thereby exhibiting excellent soot oxidation activity. SEM analysis was performed for the purpose of observing the morphology of the synthesized pyrochlore oxides, as shown in Figure 2. It can be seen that all samples mainly consisted of spherical primary particles with an estimated diameter of 50–80 nm, accompanied by a certain degree of sintering. Noticeably, small pores formed by the accumulation of basic particles can be observed over all oxides, which might vitally facilitate a gaseous oxygen reaction during catalysis. No obvious morphological changes can be identified based on the obtained SEM images of these catalysts. The features of pore distribution in detail need to be further investigated by the following N_2_ adsorption-desorption characterization.

### 3.3. N_2_ Adsorption-Desorption Characterization

N_2_ physisorption experiments were executed to obtain the direct information on the texture characteristics of the oxides. As shown in Figure S1a, all samples clearly present Type II adsorption isotherms in the IUPAC classification with an H3 hysteresis loop typical of non-porous or macroporous materials [19]. It can be seen from the BJH pore size distribution curves (see Figure S1b) that most of the pores fall in macro size range. The hysteresis loops at a high relative pressure indicate a capillary condensation of adsorbate in the macropores of the solids.

The specific surface area (*S_BET_*), total pore volume (*V_P_*) and average pore diameter (*D_P_*) of the oxides determined from the isotherms are listed in Table 1. The synthesized pyrochlore oxides exhibit relatively low specific surface areas (17.92–27.22 m^2^/g), consistent with results reported in previous studies [12,20]. One possible explanation to the poor surface areas can be that a high synthesis temperature (>700 °C) is required to form a pyrochlore phase. 

Among all oxides, LCS shows the largest specific surface area of 27.22 m^2^/g and pore volume of 0.12 cm^3^/g, which avail to supply more active sites for adsorption of reactive gas molecules. It appears that the Ca-doped samples (LCS and LCSC) exhibit greater specific surface area and extended pore morphology, indicating that calcium may act as a structural promoter [21]. All the advantages may probably increase the contact area between soot and catalysts based on the so-called “triple contact point (soot, catalyst and gas)” theory. Notwithstanding, it is worth mentioning that in addition to the structural properties, the reducibility of the components contained in the catalyst and the degree of lattice deficiency are all factors that may affect the activity of the catalyst. 

### 3.4. FT-IR

**Figure 3:**
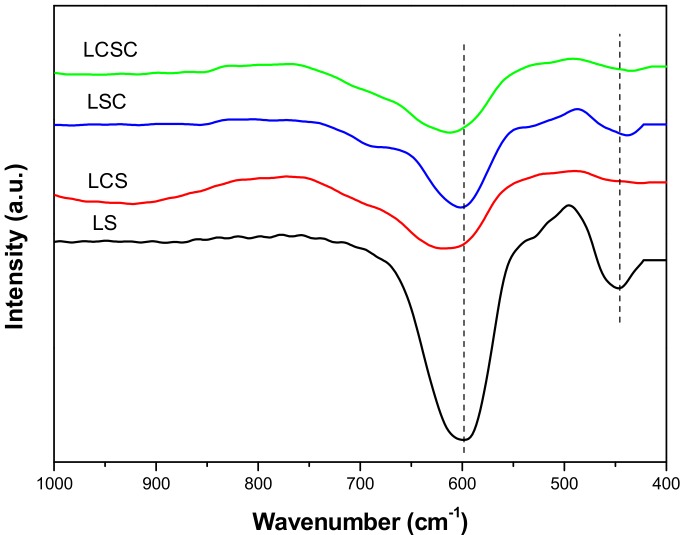
Fourier-transform infrared spectroscopy (FT-IR) spectra of La_2−x_Ca_x_Sn_2−y_Co_y_O_7_ catalysts.

For pyrochlore compounds, usually the region of interest in IR adsorption spectra is in the range of 400–1000 cm^−1^, which is ascribed to the vibrations of cations in the crystal lattice [22]. The IR spectra of the as-prepared La_2−x_Ca_x_Sn_2−y_Co_y_O_7_ samples are shown in Figure 3. For all oxides, a prominent absorption band (ν_1_) at about 600 cm^−1^ and a weaker (ν_2_) at about 450 cm^−1^ can be observed. According to the literature [23], the band at about 600 cm^−1^ is from the B–O (mainly Sn–O) stretching vibrations in the BO_6_ octahedron and the band around 450 cm^−1^ can be ascribed to the A–O′(mainly La–O) stretching vibrations. It can be seen from Figure 3 that the ν_1_ (B–O) of all doping samples slightly shift to a higher frequency compared to LS, indicating changes in B–O bond strength. It was reported that B–O bonding energy is always lower than A–O′ in pyrochlore crystallite and that the difference in the B–O bond strength would affect the release of lattice oxygens and oxygen vacancy formation [24]. Therefore, the generation of surface oxygen defects, by breaking oxygen bonds in the vicinity of the surface, may be promoted by the incorporation of Ca/Co ions.

### 3.5. H_2_-TPR

**Figure 4:**
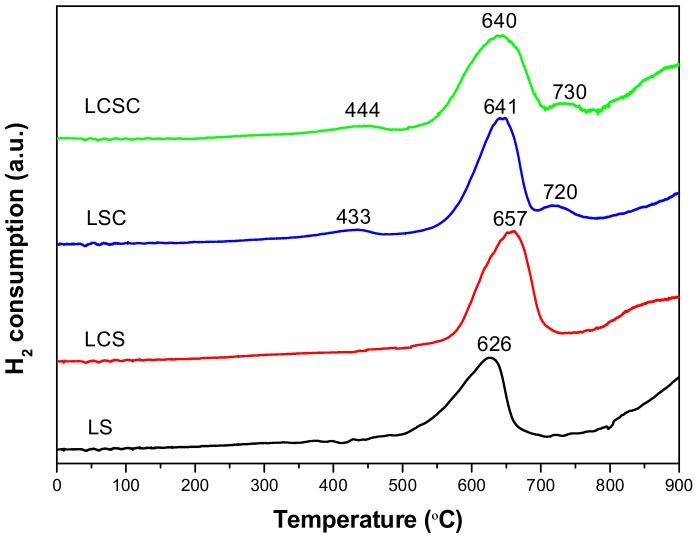
H_2_-TPR profiles of La_2−x_Ca_x_Sn_2−y_Co_y_O_7_ oxides catalysts.

Since most heterogeneous catalytic reactions occur on the surface of the catalyst, morphology, specific surface area as well as porosity are the important features that effect the oxidation reaction for the material [8,10,25]. However, from the viewpoint of pure oxidation-reduction activity, the reduction ability of a substance derived from the metal-oxygen bond is directly related to the redox activity. The reducibility of the pyrochlore oxides was characterized by the TPR technique and the TPR profiles are depicted in Figure 4. It can be seen that pure La_2_Sn_2_O_7_ shows a main hydrogen consumption peak between 500 °C and 700 °C, which corresponds to a partial reduction of Sn^4+^ to Sn^2+^. The higher temperature peak above 750 °C can be assigned to the reduction of Sn^2+^ → Sn^0^. That is, the pyrochlore structure is difficult to reduce when the temperature is below 500 °C. After the incorporation of Ca into the La_2_Sn_2_O_7_ system, the first reduction was slightly delayed. Furthermore, as Co was introduced into La_2_Sn_2_O_7_, the LSC and LCSC samples showed profiles containing two additional peaks around 440 °C and 720 °C, which evinced the stepwise reduction of cobalt in lattice (Co^3+^ to Co^2+^ and Co^2+^ to Co^0^, respectively) [26]. According to the previous report [15], it precisely is the interaction between Co and Sn in the lattice that heavily modified the redox property of the oxide by doping Co with multiple valences. However, for the LCSC sample, those two Co reduction peaks were gently shifted to a higher temperature range comparing with LSC, indicating the decreased reducibility of LCSC. In general, oxides with more readily reduced oxygen species have higher redox activity. When these catalysts are compared based on their lowest temperature reduction peaks, the reducibility decreases in the order of LSC (433 °C) > LCSC (444 °C) > LS (626 °C) > LCS (657 °C).

### 3.6. Catalyst Chemical States and Surface Oxygen Vacancies

The chemical states and the relative abundance of elements on the surface of oxides were analyzed using the XPS technique. 

Figure S2a depicts the XPS spectra of La 3d, which has the typical position (833.3–833.9 eV) and a band shape of La (III) compounds [27]. Both La 3d_3/2_ and La 3d_5/2_ show double peaks. Natile et al. [28] once explained that the bimodal was resulted from the energy loss phenomenon (“jitter” satellite) caused by the charge transfer of O 2p → La 4f or the strong final state mixing of electronic configuration. The substitution of La by Ca atoms caused a small shift in these peaks to higher binding energies, which was probably connected with a different chemical environment. On the other hand, it was clear that the doping of cobalt did not affect the peak position of La 3d. Among all oxides, LSC, and LCSC display weaker responses than LS and LCS, which is consistent with the surface atomic percentage summarized in Table 2, conveying that Co ions distort the balance state between La and surroundings, with less La exposed on the outer-layer of lattice. 

Figure S2b depicts the Sn 3d spectra of different catalysts, which has the typical position (485.5–486.0 eV) and band shape of Sn (IV) compounds [29]. It can be seen that the change of dominant position is similar to La 3d, which proves that the introduction of calcium made the lattice internal environment undergo great changes. Furthermore, consistent with the surface atomic content, the overall peak intensity of LCSC is much weaker than others, implying lower Sn exposure on the catalyst surface after the double substitution. 

Figure S2c depicts the Ca 2p patterns of LCS and LCSC. The spectra show the 2p_3/2_ and 2p_1/2_ components of the 2p level at around ~346.5 eV and ~350.5 eV with an intensity ratio of 2:1 among them, and separated by 4.0 eV due to spin orbit coupling. The binding energy values of Ca 2p in LCS and LCSC agree with the literature value [30], thus confirms that Ca is in +2 valence state in lattice.

Figure S2d shows the Co 2p patterns of LSC and LCSC. The two that are marked peak at around ~779.1 eV and ~794.4 eV due to the spin orbit split of 2p_3/2_ and 2p_1/2_, respectively. It can be noticed that in the case of LCSC, quite low signal intensity is present in the spectra, indicating that Ca ions distort the surroundings around Co ions in crystal. Nevertheless, the prominent peak of 2p_3/2_ over two samples powerfully proves that Co is in +3 valance state, as reported in previous data [28,31].

**Figure 5:**
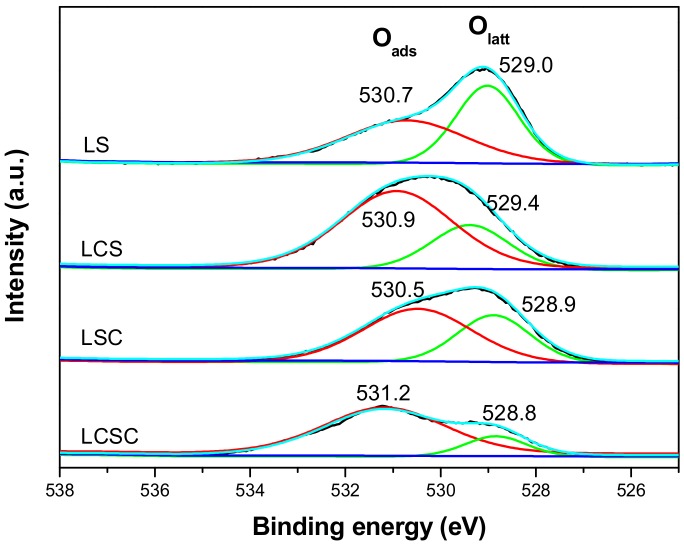
XPS spectra of O 1s region of La_2−x_Ca_x_Sn_2−y_Co_y_O_7_ catalysts.

Figure 5 exhibits the XPS spectra of O 1s that contains two oxygen signals: lattice oxygen (O_latt_: O^2−^) at 528.8–529.4 eV and surface chemisorbed oxygen (O_ads_: O^−^ or O_2_^2−^) at 530.5–531.2 eV [32]. According to the oxygen spillover mechanism, when more oxygen vacancies are generated, more molecular oxygen may be adsorbed on the oxygen vacancy forming surface adsorbed oxygen species and activated by dissociating into the reactive oxygen species, thereby accelerating the oxidation reaction [33]. Generally, the number of surface oxygen vacancies over solid oxide materials can be roughly estimated based on the ratio of O_ads_/O_latt_. Table 2 summarizes the values of O_ads_/O_latt_, decreasing in the following order: LCSC > LCS > LSC > LS, indicating the increase of oxygen vacancy after substitution. An obvious highest O_ads_/O_latt_ ratio has been obtained for the LCSC sample, indicating oxygen species are the most abundant at the surface of the dually-substituted sample. The partial substitution of La by Ca and Sn by Co simultaneously in lanthanum stannate pyrochlore structure substantially changes electron density distribution in the structure and thus oxygen vacancies are formed to keep the lattice charge balance. For the same circumstances, the oxygen vacancies of single-element-substituted samples (LCS and LSC) were increased as expected when compared to the dopant-free LS. Under an oxygen atmosphere, oxygen vacancies can effectively combine with oxygen molecules, resulting in the formation of positive holes and partially reduced oxygen, such as O_2_^2^^−^, O_2_^−^ or O^−^, which has been proposed to be a strongly electrophilic medium in oxidation reactions [34].

From the surface elements contents listed in Table 2, it can be seen that the determined value is always disagreeing with the stoichiometry ratio of each sample, indicating that the lack of an oxygen species on the pyrochlore surface is expected and a different surface element enriches the different elements’ doping catalysts. 

### 3.7. Catalytic Soot Combustion over La_2−x_Ca_x_Sn_2−y_Co_y_O_7_ Catalysts

**Figure 6:**
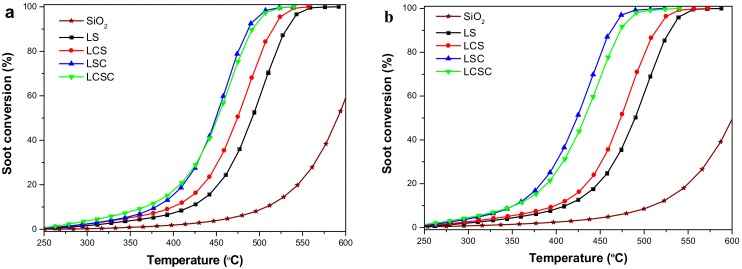

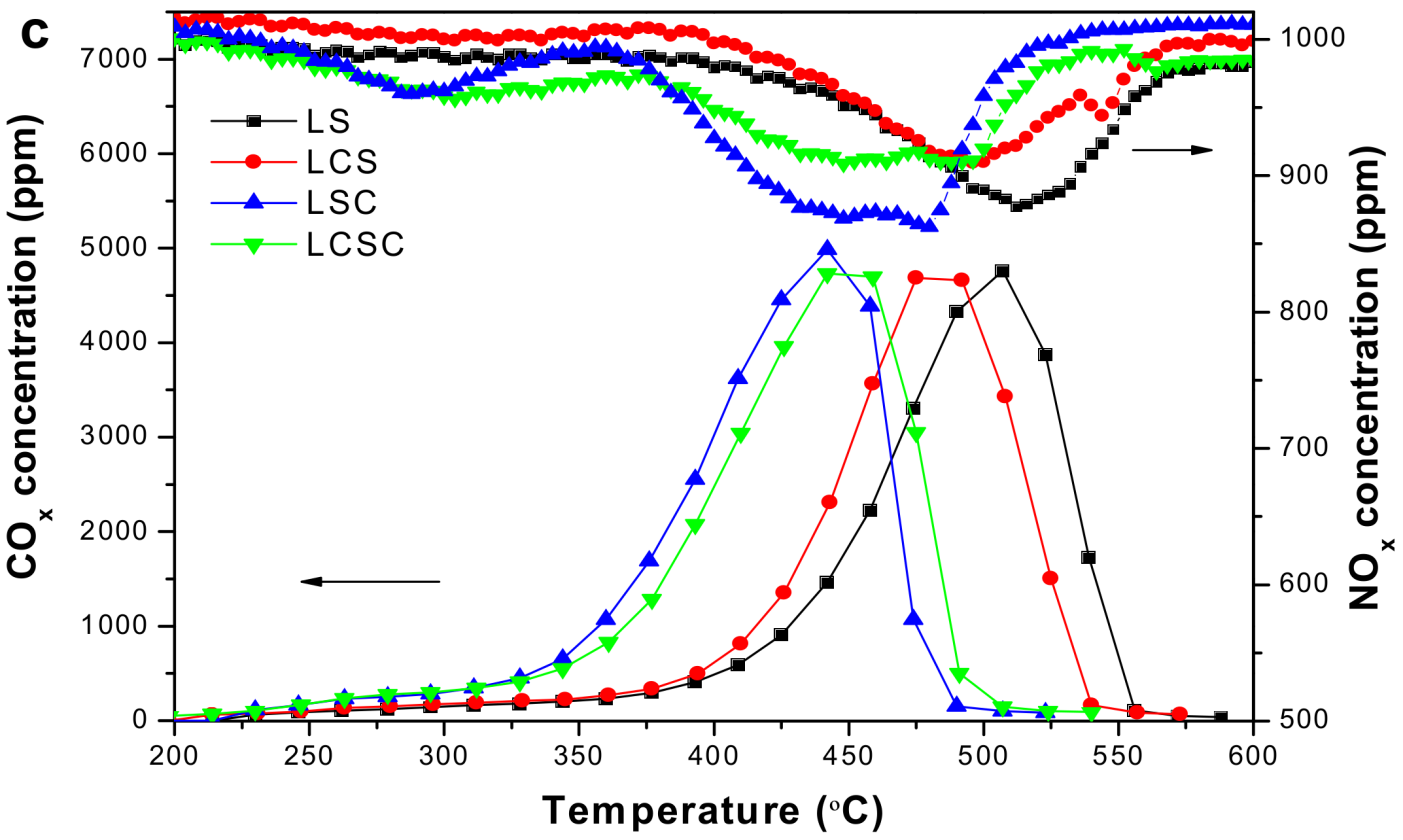
Catalytic oxidation of soot over La_2−x_Ca_x_Sn_2−y_Co_y_O_7_ oxides catalysts (**a**) 5 vol % O_2_ balanced with He; (**b**) 1000 ppm NO with 5 vol % O_2_ balanced with He; and (**c**) outlet CO_2_ and NO_x_ concentration profiles.

The performance of La_2−x_Ca_x_Sn_2−y_Co_y_O_7_ (x = 0, 0.4; y = 0, 0.2) catalysts for soot combustion in the atmosphere of O_2_ and NO + O_2_ is shown in Figure 6 and Table 3. For comparison, the result of un-catalyzed soot oxidation is also included. As shown in Figure 6a, the un-catalyzed experiment with O_2_ was performed mixing the soot with SiO_2_, and the ignition temperature was 470 °C (T_5_) while the soot was totally burnt at 633 °C (T_90_). In the presence of catalysts, the soot conversion curves shift to lower a temperature range and the CO_2_ selectivity was significantly improved (see Table 3). Pure La_2_Sn_2_O_7_ exhibits modest activity with a shift of T_5_ by 98 °C to lower temperature. When La_2_Sn_2_O_7_ was partially substituted by Ca or Co separately, the T_5_ was further decreased to 346 °C and 341 °C, respectively. 

The enhanced activity of the Ca-substituted sample could be partly ascribed to the improved contact condition between catalyst and soot and the formation of more oxygen vacancies. As shown by the previous XRD and BET results, the introduction of calcium inhibits the crystallinity of the catalyst crystals to some extent and the Ca-modified sample shows smaller particle size and bigger specific surface area, which are favorable to achieving high contacting efficiency between soot and catalyst. Furthermore, in the view of oxygen spillover, gaseous oxygen can be adsorbed on the surface oxygen vacancies and activated by dissociating into active oxygen species, which are subsequently transported through the surface oxide layer to the catalyst-soot interface, where the soot particles are oxidized into carbon oxides. Thus, oxygen vacancy can be also regarded as one kind of catalytically active site in a redox cycling [2]. Substitution of La^3+^ by lower valence Ca^2+^ leads to generating higher concentrations of oxygen vacancies, which has been confirmed by XPS study. Increasing oxygen vacancies in a LCS structure substantially increases the adsorption and activation rate of oxygen at the catalyst surface.

The electropositivity of Ca component in the catalyst may be another factor for improved catalysis behavior over the LCS sample. Luca Lietti et al. [7] once suggested that the electronegativity of alkali and alkaline-earth metals may account for their remarkable reactivity for soot removal. B. Ura et al. also stated that the beneficial electropositive nature of alkaline metal might result in a strengthening of carbon-oxygen bonds and a corresponding weakening of carbon-carbon bonds based on an electron transfer theory, and thus promoting the carbon gasification process [34]. 

The promotional effect on catalytic activity by incorporating Co is attributed to not only the increasing oxygen vacancy content originating from the substitution of Sn^4+^ by Co^3+^, but also improved reducibility. Specifically, Co doping effectively changed the strength of the B-O bond in the crystallite, making it more prone to rupture at 300–600 °C, proving that the released capacity of lattice oxygen greatly improves and the mobility of oxygen species from bulk to surface is significantly enhanced. Meanwhile, more additional active sites of Co (Co^3+^, Co^2+^) were generated during redox proceeding, as described in the TPR analysis. 

Once Ca and Co were simultaneously introduced into the structure of the La_2_Sn_2_O_7_ pyrochlore, the T_5_ was greatly decreased to 317 °C, revealing the synergistic effect between the Ca and Co cations in the lattice, which was mainly manifested in a substantial increase in oxygen vacancy concentration. Furthermore, LCSC possessed a small particle size, a suitable surface electropositivity and an excellent reducibility. All these superiorities directly determined the further decreased ignition temperature of the dually-doping sample. 

Table 3 summarizes the selectivity to CO_2_ formation (S_CO2_) for the catalytic reaction of each catalyst. It can be seen that La_2_Sn_2_O_7_ improved this value by about 50% from the non-catalytic situation. The A-site incorporation of Ca did not seem to enhance that value, whereas the introduction of Co at the B-site increased it to 96.9%, which can be further confirmed from the double-doped LCSC sample. This indicates that the Co component plays a vital role in promoting CO_2_ production in the course of soot combustion, mainly due to the high CO oxidation activity of Co_3_O_4_ as reported before [35].

Based on the NO_2_-assisted combustion mechanism, we also explored the catalytic behavior of each catalyst in the presence of NO + O_2_. As shown, the soot conversion profiles are in Figure 6b and the parameters of soot combustion are in Table 3. While nitric oxide was present, the combustion temperature of LSC and LCSC decreased more obviously than others when compared with the results in the oxygen atmosphere, which seemed to indicate that in the NO + O_2_ atmosphere these two catalysts more fully utilized NO. For the sake of clarity, Figure 6c shows the evolutions of CO_x_ and NO_x_ during the soot oxidation process. As shown, the LSC and LCSC consumed larger number of NO_x_ and the whole process showed a continuing consumption trend to it with the more CO_x_ releasing in relative low temperatures, demonstrating the fact that their surface was more likely to adsorb nitric oxide, producing nitrates or nitrites to release nitrogen dioxide to accelerate burning at the ignition stage. An obvious NO_x_ desorption peak appears at 350 °C for both LSC and LCSC, just after the onset of soot combustion, referring to the results of soot combustion in Table 3. Therefore, a large amount of NO_x_ released is believed to be caused by the decomposition of nitrate and/or nitrite species, which is promoted by the intense exothermic combustion of soot [36]. Subsequently, NO_x_ concentration dramatically decreased, demonstrating the occurrence of NO_x_ reduction by soot [37]. It can be seen that for the LS and LCS, there appears to be no NO_x_ participates into the soot ignition reaction at low temperature, but similarly a significant NO_x_ consumption peak can be observed after ignition, attributable to the reduction of NO_x_ by soot. In addition, Table 3 also lists the characteristic temperature and CO_2_ selectivity of each catalyst in the presence of NO. It is found that the presence of NO does not affect S_CO2_, indicating that CO formation may only be related to O_2_.

### 3.8. Catalytic Activities for NO Oxidation

**Figure 7:**
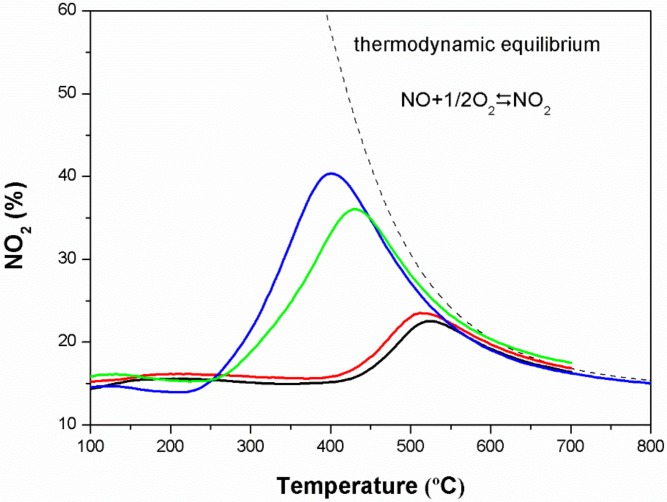
Oxidation of NO to NO_2_ in the absence of soot. NO_2_ (%) was calculated as 100 × [NO_2_]^out^/([NO]^out^ + [NO_2_]^out^).

NO_2_ can play an important role in auxiliary combustion during soot catalytic oxidation due to its greater oxidability than O_2_ [5,38]. In order to obtain information about the relationship between NO_2_ production and soot combustion for the prepared oxide catalysts that evince the NO_2_-assited mechanism, the NO oxidation experiments were carried out, and the results are depicted in Figure 7. 

According to the literature [39], the gas phase oxidation of NO to NO_2_ is almost negligible within the exhaust temperature range of diesel vehicles and is only detected above 500 °C. As can be seen from Figure 7, in the presence of the catalyst, NO can be readily oxidized to NO_2_ on all catalysts at low temperatures with about 15% of NO converted. It is worth mentioning that LCS and LS have qualitatively similar NO oxidative capacities, which mainly took place at high temperatures (maximum at 500 °C). A slightly better performance of LCS may be attributed to more active adsorption sites due to the increased specific surface area and oxygen defect concentration after introducing calcium. Moreover, the LSC and LCSC catalysts yield more NO_2_ and tend to lower temperatures, which is consistent with their catalytic effect on soot combustion. It is confirmed that the NO_2_ production by the pyrochlore oxide is improved by Co doping, which explains the superior promotion effect of LSC and LCSC for soot removal in the NO + O_2_ atmosphere. In addition, from the maximum NO_2_ generating level in the TPO profiles, the NO_2_ production capacity decreases by the following order: LSC > LCSC > LCS > LS. These results clearly indicate that the presence of the dopant permits the boost of NO_2_ formation. 

### 3.9. In Situ DRIFTS 

In the preceding section, it was confirmed that the presence of NO contributes to the oxidation of soot, that is, is based on the NO_2_-assist mechanism. In order to further investigate the catalytic oxidation of NO to NO_2_ by the Co-containing catalysts, the in situ DRIFT experiments were carried out under a NO + O_2_ gas stream from RT to 500 °C, and the surface processes have been analyzed. 

**Figure 8:**
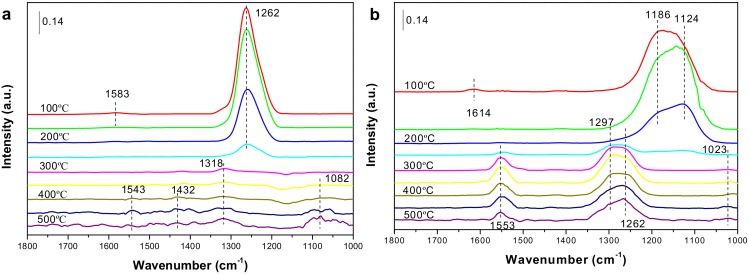
Diffuse reflection infrared Fourier transformed (DRIFT) spectra obtained on (**a**) LSC and (**b**) LCSC catalysts exposed to 1000 ppm NO/5% O_2_/He with the increase of temperature.

As shown in Figure 8a, the intermediates formed by the adsorption are mainly chelating nitrites (1262 cm^−1^) on LSC at low temperatures (<300 °C) accompanied by initial bidentate nitrates (1583 cm^−1^), which disappeared immediately after 100 °C [9,40]. As the temperature increases, the band intensity for above nitrites species declines gradually and disappears at 300 °C with simultaneous forming of trace nitrates, including monodentate nitrates (1082, 1432 cm^−1^) and bidentate nitrates (1318, 1543 cm^−1^) [9,41], which are extremely stable at high temperatures. Considering the NO-TPO results, the trend of NO_2_ production correlates well with that of the IR intensity of nitrites versus temperature, which well illustrates that the NO_2_ involved in the oxidation of soot originates from the decomposition of nitrites due to inferior thermostability.

The formation of nitrates is advanced by about 50 °C on LCSC, as depicted in Figure 8b, implying that the co-existence of Ca and Co results in a more facile formation of nitrate species from nitrites. Similar to the LSC sample, at low temperatures (<250 °C) the adsorbate species on the catalyst are mainly monodentate nitrites (1124 cm^−1^) and bridged bidentate nitrites (1186 cm^−1^), accompanied by a small amount of bridged bidentate nitrates (1614 cm^−1^) [42], appears at about 100 °C and then immediately disappears. After heating to 250 °C, the catalyst only reserves trace more-stable bidentate nitrates (1025, 1297 cm^−1^) and monodentate nitrates (1262, 1553 cm^−1^) [43]. It is noteworthy that compared with LSC, the intensity of nitrate species adsorbed on the LCSC catalyst is stronger in the higher temperature range. This result is consistent with the previous NO oxidation, that is, the calcium dopant enhances the stability of nitrate species on the catalyst, which leads to less releasing NO_2_ in the NO oxidation process. Furthermore, several different types of nitrites/nitrates appear on these two samples, indicating different active sites are present as Ca is introduced.

## 4. Conclusions

In this study, a series of lanthanum-tin pyrochlore oxides with A/B sites substituted by Ca/Co were successfully prepared for catalytic combustion of soot particulates. Dually-substitution of calcium and cobalt improved the structural properties and redox properties of the catalyst. Simultaneously, LCSC possessed a favorable electropositivity. Therefore, LCSC presents a much lower ignition temperature with an overall high performance towards soot combustion, revealing the synergistic effect of Ca and Co in the crystal. In addition, doping allowed the oxygen vacancy concentration on the catalyst surface to be enhanced, facilitating the adsorption activation of O_2_/NO during the catalytic process. Furthermore, two main periods of NO adsorption on the catalysts are observed by the DRIFT technique: the low temperature stage (<250–300 °C) and the high temperature stage (>300 °C). Indeed, it reveals that the low temperature adsorption period is mainly associated with weakly adsorbed nitrite species that are responsible for the production of NO_2_ participating in the ignition period of soot combustion.

## Figures and Tables

**Table 1:** Structural information of the pyrochlore catalysts.

Samples	2θ (°) ^a^	*a* (Å) ^b^	*Xs* (nm) ^c^	*S_BET_* (m^2^/g) ^d^	*V_p_* (cm^3^/g) ^e^	*D_p_* (nm) ^f^
LS	28.88	10.6965 ± 0.0011	22.2	19.59	0.08	17.91
LCS	28.92	10.6773 ± 0.0021	12.1	27.22	0.12	18.21
LSC	28.96	10.6863 ± 0.0020	21.4	17.92	0.06	13.36
LCSC	28.96	10.6711 ± 0.0038	8.9	21.18	0.10	18.17

^a^ 222 crystal face; ^b^ Lattice constant calculated from X-ray powder diffraction (XRD) characteristic peaks of samples; ^c^ Average crystallite size calculated from the 222 crystal face by XRD; ^d^ Brunauer–Emmett–Teller (BET) specific surface area; ^e^ Total pore volume; ^f^ Average pore diameter.

**Table 2:** X-ray photoelectron spectroscopy (XPS) quantitative analysis results of catalysts.

Samples	La (at %)	Sn (at %)	Theoretical La/Sn	Practical La/Sn	Ca (at %)	Co (at %)	O_ads_/O_latt_
LS	14.05	21.22	1.00	0.66	-	-	1.02
LCS	14.17	17.24	0.8	0.82	0.66	-	2.44
LSC	11.56	20.7	1.11	0.56	-	1.17	1.66
LCSC	11.31	12.12	0.89	0.93	0.98	0.88	4.25

**Table 3:** Catalytic performances of oxides for soot removal.

Samples	Soot + O_2_				Soot + NO + O_2_			
	T_5_ (°C)	T_50_ (°C)	T_90_ (°C)	S_CO2_ (%)	T_5_ (°C)	T_50_ (°C)	T_90_ (°C)	S_CO2_ (%)
Blank	470	590	633	37.7	460	600	647	49.1
LS	372	492	531	86.6	360	489	530	85.8
LCS	346	474	516	85.1	343	473	514	86.8
LSC	341	449	487	96.9	313	424	463	96.2
LCSC	317	451	491	96.4	305	432	473	95.7

## Supplementary Materials

The following are available online at http://www.mdpi.com/1996-1944/11/5/653/s1.

## References

[1] Zhang G., Zhao Z., Xu J., Zheng J., Liu J., Jiang G., Duan A., He H. (2011). Comparative study on the preparation, characterization and catalytic performances of 3DOM Ce-based materials for the combustion of diesel soot. Appl. Catal. B Environ..

[2] Jampaiah D., Venkataswamy P., Tur K.M., Ippolito S.J., Bhargava S.K., Reddy B.M. (2015). Effect of mnoxloading on structural, surface, and catalytic properties of CeO_2_-mnoxmixed oxides prepared by sol-gel method. Z. Anorg. Allg. Chem..

[3] Russo N., Fino D., Saracco G., Specchia V. (2008). Promotion effect of au on perovskite catalysts for the regeneration of diesel particulate filters. Catal. Today.

[4] Lee C., Park J.-I., Shul Y.-G., Einaga H., Teraoka Y. (2015). Ag supported on electrospun macro-structure CeO_2_ fibrous mats for diesel soot oxidation. Appl. Catal. B Environ..

[5] Fino D., Russo N., Saracco G., Specchia V. (2006). Catalytic removal of nox and diesel soot over nanostructured spinel-type oxides. J. Catal..

[6] Guo X., Meng M., Dai F., Li Q., Zhang Z., Jiang Z., Zhang S., Huang Y. (2013). Nox-assisted soot combustion over dually substituted perovskite catalysts La_1−x_K_x_Co_1−y_Pd_y_O_3−δ_. Appl. Catal. B Environ..

[7] Castoldi L., Matarrese R., Lietti L., Forzatti P. (2009). Intrinsic reactivity of alkaline and alkaline-earth metal oxide catalysts for oxidation of soot. Appl. Catal. B Environ..

[8] Venkataswamy P., Jampaiah D., Rao K.N., Reddy B.M. (2014). Nanostructured Ce_0.7_Mn_0.3_O_2−δ_ and Ce_0.7_Fe_0.3_O_2−δ_ solid solutions for diesel soot oxidation. Appl. Catal. A Gen..

[9] Lin F., Wu X., Weng D. (2011). Effect of barium loading on CuO_x_–CeO_2_ catalysts: NO_x_ storage capacity, NO oxidation ability and soot oxidation activity. Catal. Today.

[10] Oliveira C.F., Garcia F.A.C., Araújo D.R., Macedo J.L., Dias S.C.L., Dias J.A. (2012). Effects of preparation and structure of cerium-zirconium mixed oxides on diesel soot catalytic combustion. Appl. Catal. A Gen..

[11] Zahir M.H., Suzuki T., Fujishiro Y., Awano M. (2009). Hydrothermal synthesis of Sr–Ce–Sn–Mn–O mixed oxidic/stannate pyrochlore and its catalytic performance for NO reduction. Mater. Chem. Phys..

[12] Peng H., Xu J., Tian J., Liu Y., He Y., Tan J., Xu X., Liu W., Zhang N., Wang X. (2016). Mesoporous Y_2_Sn_2_O_7_pyrochlore with exposed (111) facets: An active and stable catalyst for CO oxidation. RSC Adv..

[13] Modeshia D.R., Walton R.I. (2010). Cheminform abstract: Solvothermal synthesis of perovskites and pyrochlores: Crystallization of functional oxides under mild conditions. Chem. Soc. Rev..

[14] Teraoka Y., Torigoshi K.I., Yamaguchi H., Ikeda T., Kagawa S. (2000). Direct decomposition of nitric oxide over stannate pyrochlore oxides: Relationship between solid-state chemistry and catalytic activity. J. Mol. Catal. A Chem..

[15] Cheng J., Wang H., Hao Z., Wang S. (2008). Catalytic combustion of methane over cobalt doped lanthanum stannate pyrochlore oxide. Catal. Commun..

[16] Shangguan W.F., Teraoka Y., Kagawa S. (1998). Promotion effect of potassium on the catalytic property of CuFe_2_O_4_ for the simultaneous removal of NO_x_ and diesel soot particulate. Appl. Catal. B Environ..

[17] Lv M., Guo X., Wang Z., Wang L., Li Q., Zhang Z. (2016). Synthesis and characterization of Co–Al–Fe nonstoichiometric spinel-type catalysts for catalytic CO oxidation. RSC Adv..

[18] Wu S., Zhang L., Wang X., Zou W., Cao Y., Sun J., Tang C., Gao F., Deng Y., Dong L. (2015). Synthesis, characterization and catalytic performance of FeMnTiO_x_ mixed oxides catalyst prepared by a CTAB-assisted process for mid-low temperature NH_3_-SCR. Appl. Catal. A Gen..

[19] Wang Z., Zhu H., Ai L., Liu X., Lv M., Wang L., Ma Z., Zhang Z. (2016). Catalytic combustion of soot particulates over rare-earth substituted Ln_2_Sn_2_O_7_ pyrochlores (Ln = La, Nd and Sm). J. Colloid Interface Sci..

[20] Fang X., Zhang X., Guo Y., Chen M., Liu W., Xu X., Peng H., Gao Z., Wang X., Li C. (2016). Highly active and stable Ni/Y_2_Zr_2_O_7_ catalysts for methane steam reforming: On the nature and effective preparation method of the pyrochlore support. Int. J. Hydrog. Energy.

[21] Kim C.H., Qi G., Dahlberg K., Li W. (2010). Strontium-doped perovskites rival platinum catalysts for treating nox in simulated diesel exhaust. Science.

[22] López-Suárez F.E., Bueno-López A., Illán-Gómez M.J., Trawczynski J. (2014). Potassium-copper perovskite catalysts for mild temperature diesel soot combustion. Appl. Catal. A Gen..

[23] Li K.W., Zhang T.T., Wang H., Yan H. (2006). Low-temperature synthesis and structure characterization of the serials Y_2−δ_Bi_δ_Sn_2_O_7_ (δ = 0–2.0 δ = 0–2.0 mathcontainer loading mathjax) nanocrystals. J. Solid State Chem..

[24] Petit C., Kaddouri A., Libs S., Kiennemann A., Rehspringer J.L., Poix P. (1993). Bond energy effects in methane oxidative coupling on pyrochlore structures. J. Catal..

[25] Bensaid S., Russo N., Fino D. (2013). CeO_2_ catalysts with fibrous morphology for soot oxidation: The importance of the soot–catalyst contact conditions. Catal. Today.

[26] Liu J., Zhao Z., Xu J., Xu C., Duan A., Jiang G., He H. (2011). The highly active catalysts of nanocomposite K-Co-CeO_2_ for soot combustion. Chem. Commun..

[27] Pecchi G., Dinamarca R., Campos C.M., Garcia X., Jimenez R., Fierro J.L.G. (2014). Soot oxidation on silver-substituted LaMn_0.9_Co_0.1_O_3_ perovskites. Ind. Eng. Chem. Res..

[28] Yao W., Wang R., Yang X. (2009). LaCo_1−x_Pd_x_O_3_ perovskite-type oxides: Synthesis, characterization and simultaneous removal of NO_x_ and diesel soot. Catal. Lett..

[29] Tian J., Peng H., Xu X., Liu W., Ma Y., Wang X., Yang X. (2015). High surface area La_2_Sn_2_O_7_ pyrochlore as a novel, active and stable support for Pd for CO oxidation. Catal. Sci. Technol..

[30] Asuvathraman R., Gnanasekar K.I., Clinsha P.C., Ravindran T.R., Govindan Kutty K.V. (2015). Investigations on the charge compensation on Ca and U substitution in CePO_4_ by using XPS, XRD and raman spectroscopy. Ceram. Int..

[31] Natile M.M., Ugel E., Maccato C., Glisenti A. (2007). LaCoO_3_: Effect of synthesis conditions on properties and reactivity. Appl. Catal. B Environ..

[32] Tang X., Li Y., Huang X., Xu Y., Zhu H., Wang J., Shen W. (2006). MnOx–CeO_2_ mixed oxide catalysts for complete oxidation of formaldehyde: Effect of preparation method and calcination temperature. Appl. Catal. B Environ..

[33] Piumetti M., Bensaid S., Russo N., Fino D. (2016). Investigations into nanostructured ceria–zirconia catalysts for soot combustion. Appl. Catal. B Environ..

[34] Ura B., Trawczyński J., Kotarba A., Bieniasz W., Illán-Gómez M.J., Bueno-López A., López-Suárez F.E. (2011). Effect of potassium addition on catalytic activity of SrTiO_3_ catalyst for diesel soot combustion. Appl. Catal. B Environ..

[35] Jia C.J., Schwickardi M., Weidenthaler C., Schmidt W., Korhonen S., Weckhuysen B.M., Schüth F. (2011). Co_3_O_4_-SiO_2_ nanocomposite: A very active catalyst for Co oxidation with unusual catalytic behavior. J. Am. Chem. Soc..

[36] Li Z., Meng M., Zha Y., Dai F., Hu T., Xie Y., Zhang J. (2012). Highly efficient multifunctional dually-substituted perovskite catalysts La_1−x_K_x_Co_1−y_Cu_y_O_3−δ_ used for soot combustion, NO_x_ storage and simultaneous NOx-soot removal. Appl. Catal. B Environ..

[37] Li Q., Meng M., Tsubaki N., Li X., Li Z., Xie Y., Hu T., Zhang J. (2009). Performance of k-promoted hydrotalcite-derived comgalo catalysts used for soot combustion, nox storage and simultaneous soot–nox removal. Appl. Catal. B Environ..

[38] Li Q., Meng M., Zou Z.Q., Li X.G., Zha Y.Q. (2009). Simultaneous soot combustion and nitrogen oxides storage on potassium-promoted hydrotalcite-based comgalo catalysts. J. Hazard. Mater..

[39] Hernández-Giménez A.M., Xavier L.P.d.S., Bueno-López A. (2013). Improving ceria-zirconia soot combustion catalysts by neodymium doping. Appl. Catal. A Gen..

[40] Wu X., Lin F., Xu H., Weng D. (2010). Effects of adsorbed and gaseous NO_x_ species on catalytic oxidation of diesel soot with MnO_x_–CeO_2_ mixed oxides. Appl. Catal. B Environ..

[41] Yang G., Li Y., Men Y. (2015). Synergistic catalysis effect of mn-promoted BaAl_2_O_4_ catalysts on catalytic performance for soot combustion. Catal. Commun..

[42] Sedlmair C. (2003). Elementary steps of nox adsorption and surface reaction on a commercial storage–reduction catalyst. J. Catal..

[43] Liu S., Wu X., Weng D., Li M., Ran R. (2015). Roles of acid sites on Pt/H-ZSM5 catalyst in catalytic oxidation of diesel soot. ACS Catal..

