# Supplementary material for: Catalytic Oxidation of Soot on a Novel Active Ca-Co Dually-Doped Lanthanum Tin Pyrochlore Oxide
Source: Materials (Basel). 2018 Apr 24;11(5):653. doi: 10.3390/ma11050653 (PMC5978030; doi:10.3390/ma11050653)
Supplement: Supplementary file 1 [file materials-11-00653-s001.zip › materials-284565-SI.pdf]

Catalytic oxidation of soot on a novel active Ca-Co dually-doped lanthanum tin pyrochlore oxide

Lijie Ai, Zhongpeng Wang*, Chenchen Cui, Wei Liu, Liguo Wang

School of Resources and Environment, Key Laboratory of Water Resources and Environmental Engineering in Universities of Shandong, University of Jinan, 336 Nanxinzhuang West Road, Jinan 250022, China; ailj20160202@163.com (L.A.); 15053102308@163.com (C.C.); stu_liuw@ujn.edu.cn (W.L.); chm_wanglg@ujn.edu.cn (L.W.)

* Correspondence: chm_wangzp@ujn.edu.cn; Tel.: +86-531-82769233

4 pages (including this page):

Figure S1-S2

Figure S1.

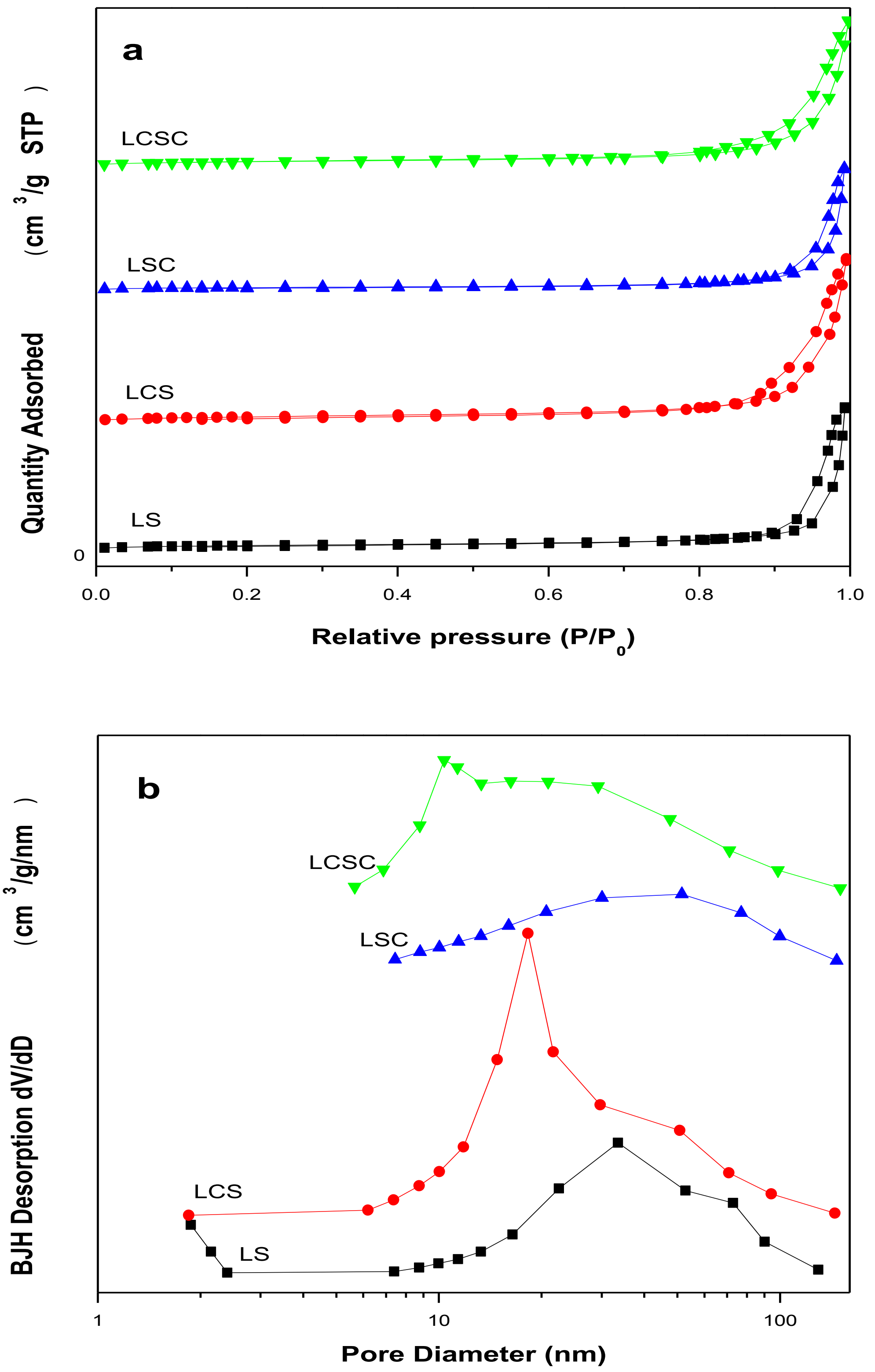

Figure S1. Nitrogen adsorption-desorption isotherms (a) and pore size distributions (b) of $La_{2-x}Ca_xSn_{2-y}Co_yO_7$ catalysts.

Figure S2.

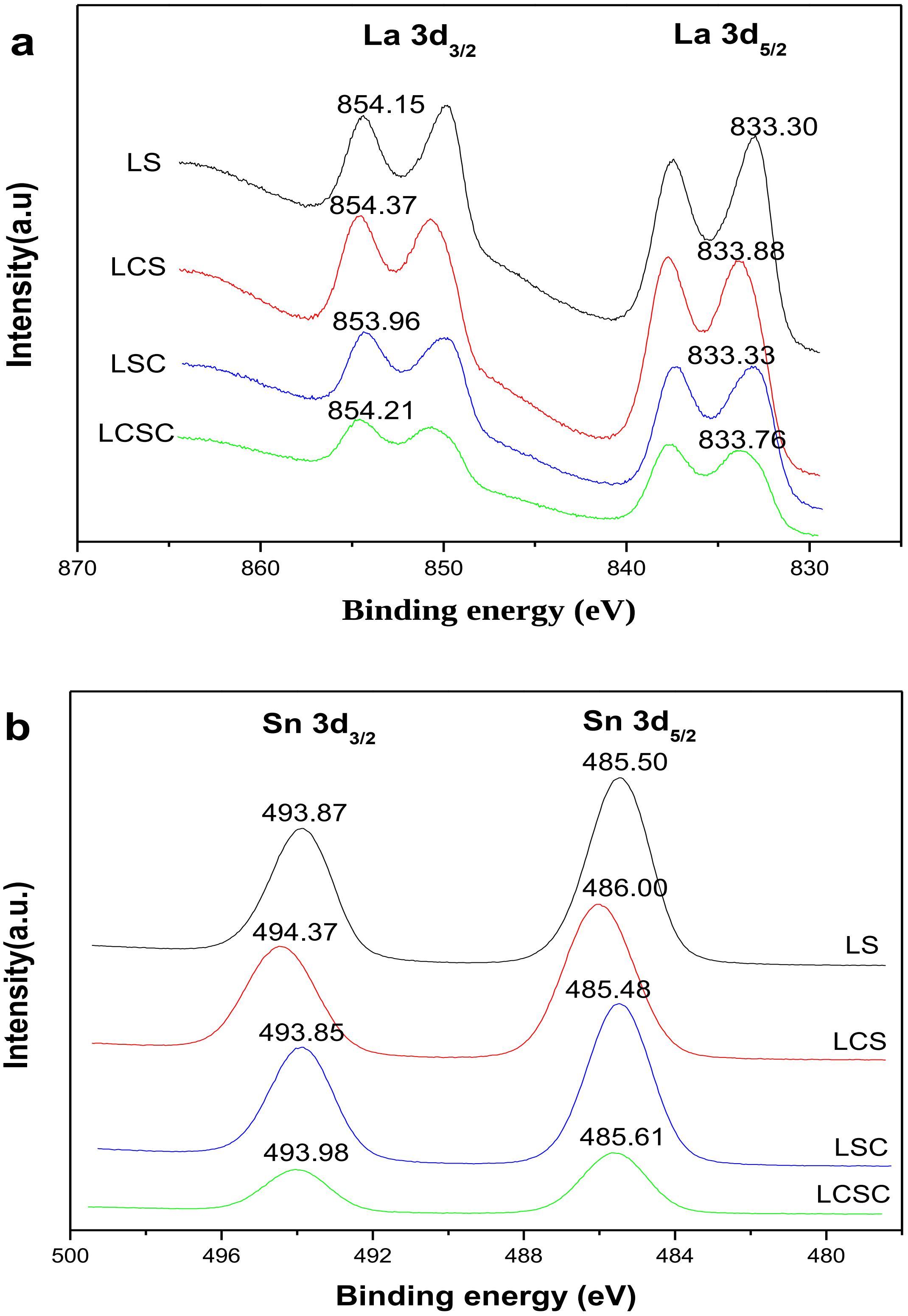

a
La 3d3/2
La 3d5/2
854.15
833.30
LS
854.37
LCS
833.88
853.96
LSC
833.33
854.21
LCSC
833.76
Intensity(a.u)
870
860
850
840
830
Binding energy (eV)
b
Sn 3d3/2
Sn 3d5/2
485.50
493.87
486.00
LS
494.37
485.48
LCS
493.85
485.61
LSC
493.98
LCSC
Intensity(a.u.)
500
496
492
488
484
480
Binding energy (eV)

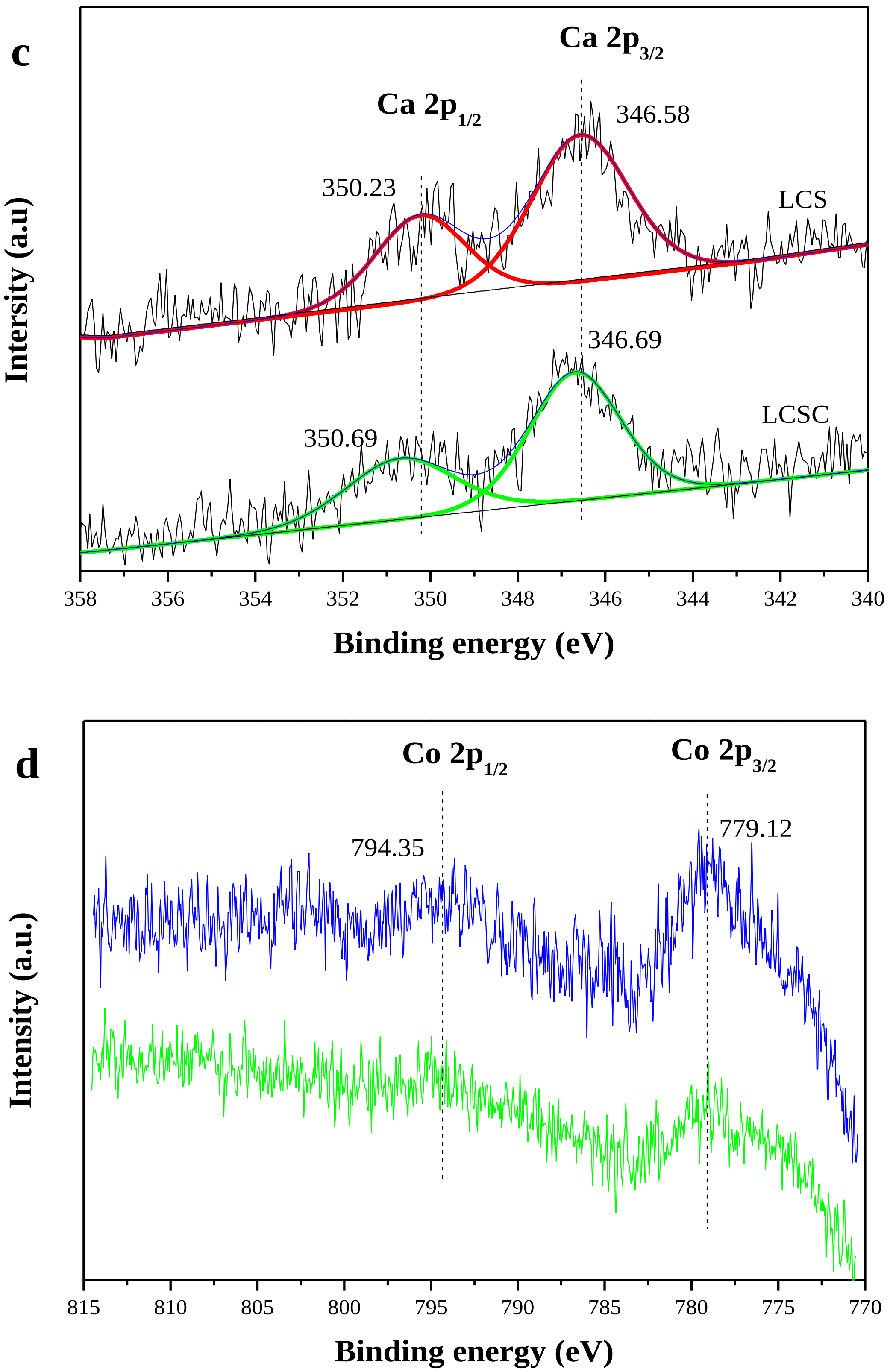

Figure S2. XPS spectra of (a) La 3d (b) Sn 3d (c) Ca 2p (d) Co 2p region of the obtained catalysts
